# Theoretical basis, application, reliability, and sample size estimates of a Meridian Energy Analysis Device for Traditional Chinese Medicine Research

**DOI:** 10.6061/clinics/2017(04)10

**Published:** 2017-04

**Authors:** Ming-Yen Tsai, Shih-Yu Chen, Chung-Chun Lin

**Affiliations:** I Department of Chinese Medicine, Kaohsiung Chang Gung Memorial Hospital and Chang Gung University College of Medicine, Kaohsiung, Taiwan; II School of Chinese Medicine for Post Baccalaureate, I-Shou University College of Medicine, Kaohsiung, Taiwan

**Keywords:** Meridian Analysis, Electrical Conductance, Application, Traditional Chinese Medicine

## Abstract

**OBJECTIVES:**

The Meridian Energy Analysis Device is currently a popular tool in the scientific research of meridian electrophysiology. In this field, it is generally believed that measuring the electrical conductivity of meridians provides information about the balance of bioenergy or Qi-blood in the body.

**METHODS AND RESULTS:**

PubMed database based on some original articles from 1956 to 2014 and the authoŕs clinical experience. In this short communication, we provide clinical examples of Meridian Energy Analysis Device application, especially in the field of traditional Chinese medicine, discuss the reliability of the measurements, and put the values obtained into context by considering items of considerable variability and by estimating sample size.

**CONCLUSION:**

The Meridian Energy Analysis Device is making a valuable contribution to the diagnosis of Qi-blood dysfunction. It can be assessed from short-term and long-term meridian bioenergy recordings. It is one of the few methods that allow outpatient traditional Chinese medicine diagnosis, monitoring the progress, therapeutic effect and evaluation of patient prognosis. The holistic approaches underlying the practice of traditional Chinese medicine and new trends in modern medicine toward the use of objective instruments require in-depth knowledge of the mechanisms of meridian energy, and the Meridian Energy Analysis Device can feasibly be used for understanding and interpreting traditional Chinese medicine theory, especially in view of its expansion in Western countries.

Scientific publications utilizing Meridian Energy Analysis Devices (MEADs) as an index of meridian energy investigation are increasing in number year by year. It would therefore seem logical to conclude that the many researchers who publish scholarly articles using such devices are fully aware of their non-invasive nature and cost-effectiveness, two desirable qualities of any clinical measurement. Recent reviews of the literature demonstrate that no adequate consensus on either of these qualities has been fully reached 1,2.

According to MEAD Theory, developed by Dr. Yoshio Nakatani in the 1950s, the right and left side of the body respectively have 12 meridian lines. As shown in Figures 1A and 1B, MEAD can reflect the conditions of certain organs through analysis of the symmetrical Yuan points on the wrists and ankles and comparison of their mutual relations and changes with micro-electrical currents to represent the physiological and pathological phenomena of the relevant meridians 3,4. The MEAD usually indicates constant values in the human body in the absence of external stimulation, visceral abnormalities, or diseases. The device is basically an amperometer employing a DC voltage of 12 V with output current of 0-200 uA. An electrical current between two acupoints of larger than 90 μA or smaller than 50 μA represents an excess or deficiency syndrome in the meridian, respectively. The procedure of the MEAD is similar to equipment described in our previous study 5. The examination room should be bright, quiet, and refreshing, with a comfortable ambient temperature. The participants should refrain from engaging in physical activity, using tobacco, ingesting caffeine, or eating for at least one hour before clinical assessment. Before the assessment begins, they must remove all metal materials or body ornaments and lie flat to rest for 15 minutes. One metal cylinder is held in the left hand of the participant. The other, a spring-loaded probe, is moistened with 5% saline and, after sterilization, applied sequentially to the 24 Yuan acupoints: Lung (Taiyuan, LU9), Pericardium (Daling, PC7), Heart (Shenmen, HT7), Small Intestine (Wangu, SI4), Triple Energizer (Yangchi, TE4), Large Intestine (Hegu, LI5), Spleen (Taibai, SP3), Liver (Taichong, LR3), Kidney (Taixi, KI3), Urinary Bladder (Jinggu, BL65), Gallbladder (Qiuxu, GB40), and Stomach (Chongyang, ST42). (Figure 2) After 2-3 seconds of 12 g/cm^2^ pressure for each point, the device is automatically interrupted, and the average value is recorded on a computerized system.

The application of MEAD is of high clinical interest not only in the objective assessment of traditional Chinese medicine (TCM) therapy and related sympathetic conditions but also in predicting the meridian flow of the corresponding organs. In 2012, Lin et al. used soft cupping with or without additional laser acupuncture and a MEAD for patients with low back pain 6. The results showed that pain scores were effectively alleviated after both treatments at the BL40. From observing the variations in the MEAD values of the Bladder Meridian, the authors concluded that soft cupping and laser acupuncture represent different purge and reinforcing characteristics in clinical practice. In a study by Hsu, healthy volunteers received short-term electrical acupuncture on the BL15, and the results revealed that a decrease in sympathetic activity after the intervention could be quantified with MEAD values 7. Another study showed that one measure of electrical conductance, the index of sympathovagal balance plus the calculus size in radiography, could be used as a valuable supplementary diagnostic method for selective urological intervention in patients with acute renal colic 8. In 2011, Huang et al. used a MEAD to detect the energy distribution of the meridian system after immediate glucose ingestion in different physical conditions 9. The findings suggested that the glucose concentration in the blood was rapidly consumed and that the transformation in meridian energy was difficult to present in “Qi” vacuity groups after glucose intake. More recently, our own report also supported the verifiability of the promoting effect of coffee consumption on a specific meridian channel 5. In that study, however, the indexes of the meridians did not provide sufficient evidence for analysis of autonomic response.

When the sympathetic activity in the patient's body is high, increased intradermal acetylcholine stimulates the secretions of sweat glands, which in turn increases skin conductance and decreases resistance. In other words, the function of peripheral autonomic innervation can be indirectly evaluated by measuring the electrical conductivity of acupoints. However, many technical factors can influence skin electrical impedance, including the size of the electrode, its contact time, and the amount of pressure it places on the skin, as well as the accuracy of the acupoint location, the bias of the control environment and, importantly, the degree of skin moisture 10,11. The variability in measurement caused by these factors casts doubt on the reliability of the measurement of skin conductance. This doubt is the reason why few references citing the validity of devices for such measurement appear in academic textbooks or high-quality, refereed journals.

Regarding the electrical activity at acupoints, Colbert et al. 2 assessed the reliability of MEAD measurement in an unrestricted-designed investigation for any diagnostic or medical condition in patients. Their study provided much useful information for the reader to use in determining the value of MEAD results in terms of retest reliability. They also raised the question of whether MEAD could be reliably used for diagnosis and/or therapeutic monitoring in the TCM field. The paper evaluates the reliability of selected studies and suggests a detailed procedure and methodology, as well as theoretical sample size requirements needed during future empirical investigations. A previous review 1 provided findings highly consistent with those of Colbert et al. Both studies concluded that the reliability of most electrical devices for the detection of meridian energy may appear low, or even unsatisfactory, for even the better-performing indices often double or halve the values from test to retest.

Although some studies quoted the earlier data of the device, the reproducibility is 93.2%, as reported by Nakatani 3, supporting the use of MEAD 12,13. Assessing the reliability of MEAD in this way and the creation of blinding designs is challenging. To build consistency, skilled operators in possession of adequate information regarding the use of such instruments are necessary. However, the potential of subconscious bias from the operators may decrease the validity of MEAD measurement. For these reasons, proper preparation and steps are important to ensure consistent and reliable of MEAD measurement, as with any medical measurement device. In fact, experimental errors can be carefully controlled by operators, and the correlation coefficient can be used to confirm the stability of MEAD values 5,14. Newer MEAD machines also provide automatic digital calibration systems to help operators to exactly control the confounding factors. As shown in Figure 1C, when contacting the metal clip with the probe produces electrical current up to 200 uA, it means that machine calibration is successful. MEAD is currently a convenient tool for TCM research, especially in the field of meridian electrophysiology.

To conclude, the assessment of TCM using objective modern instruments is just beginning. Many more rigorous studies are required in order to prove—or discredit—the value of MEAD measurement, which is largely ignored in the West. As illustrated in the article by Colbert et al. 2, such studies may also offer scientists well-reasoned items for both standardized methodology and design.

## AUTHOR CONTRIBUTIONS

All authors participated in the writing and discussed the final text.

## Figures and Tables

**Figure 1:**
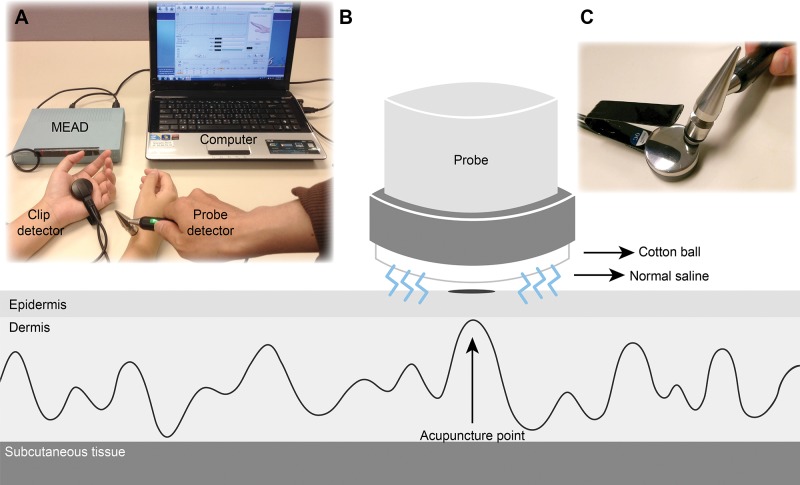
Diagram of the schematic meridian energy analysis device (MEAD). Figures 1A and 1B: The experimental setup of the MEAD Instrument and electric current measurement around the acupoints. Figure 1C: A simple way to confirm the accuracy of MEAD using automatic digital calibration systems.

**Figure 2:**
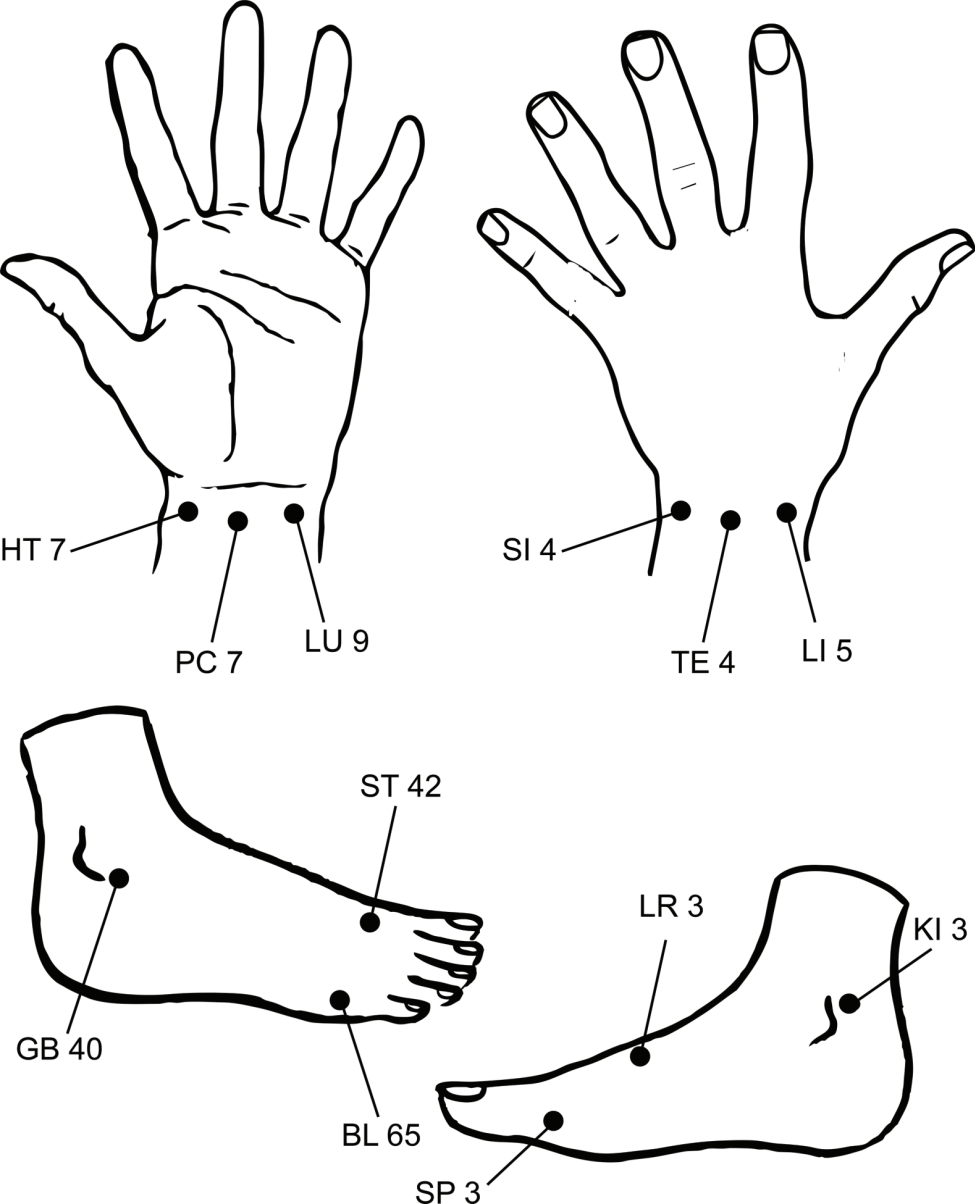
The 24 MEAD measuring points on the hands and feet according to traditional Chinese medicine theory. (LU: Lung; PC Pericardium; HT: Heart; SI: Small Intestine; TE: Triple Energizer; LI: Large Intestine; SP: Spleen; LR: Liver; KI: Kidney; BL: Urinary Bladder; GB: gallbladder; ST: Stomach).
